# Bioequivalence of a single dose of two palbociclib formulations in healthy Chinese subjects under fasting conditions: a two-period crossover study with rabeprazole pre-treatment

**DOI:** 10.3389/fphar.2025.1722151

**Published:** 2025-11-24

**Authors:** Wanjun Bai, Shuo Shi, Deliang Yin, Caihui Guo, Haojing Song, Yiting Hu, Jin Gao, Bo Qiu, Xueyuan Zhang, Lusha Bi, Huizhen Wu, Zhanjun Dong

**Affiliations:** 1 Department of Pharmacy, Hebei General Hospital, Hebei Key Laboratory of Clinical Pharmacy, Shijiazhuang, Hebei, China; 2 College of Pharmacy, Hebei Medical University, Shijiazhuang, China; 3 Beijing Kangchuanglian Biopharmaceutical Technology Research Co., Ltd., Beijing, China; 4 Shanghai Innovstone Therapeutics Limited, Shanghai, China; 5 CSPC Ouyi Pharmaceutical Co., Ltd., Shijiazhuang, China

**Keywords:** palbociclib, acid-reducing agent, pharmacokinetics, bioequivalence, safety

## Abstract

**Objectives:**

This study assessed the pharmacokinetics, safety, and bioequivalence of generic and original palbociclib tablets in healthy Chinese subjects under fasting conditions with rabeprazole pre-treatment.

**Methods:**

This was a single-dose, randomized, open-label, two-period crossover bioequivalence study conducted under fasting conditions with rabeprazole pre-treatment. In each trial, healthy Chinese subjects received 40 mg oral rabeprazole enteric-coated tablets once daily before breakfast for 6 days. Following an overnight fast of at least 10 h, they took the seventh dose of rabeprazole and maintained fasting. They then received a single 125 mg oral dose of either the test or reference palbociclib tablet, followed by a 14-day washout interval between periods. Blood samples were collected from 0 to 96 h post-dose in each period, and palbociclib plasma concentrations were determined using a validated method. The primary pharmacokinetic parameters were calculated using the non-compartmental method. The geometric mean ratios of the two formulations and the corresponding 90% confidence intervals were acquired for bioequivalence analysis. The safety of both formulations was also evaluated.

**Results:**

The 90% confidence intervals for the primary pharmacokinetic parameters of C_max_ (84.53%–91.72%), AUC_0-t_ (87.81%–92.49%), and ${\text{AUC}}_{0‐\infty }$ (87.59%–92.03%) all fell within the 80.00%–125.00% bioequivalence range. No serious adverse events occurred during the study.

**Conclusion:**

The trial confirmed that the pharmacokinetic parameters of the generic and original palbociclib tablets were bioequivalent in healthy Chinese subjects under fasting conditions with rabeprazole pre-treatment. Both formulations were safe and well tolerated.

**Clinical Trial Registration:**

http://www.chinadrugtrials.org.cn, identifier CTR20232617; https://www.chictr.org.cn, identifier ChiCTR2400084355.

## 1 Introduction

Breast cancer is the most common malignant tumor in women. According to GLOBOCAN data, there were approximately 2.3 million new breast cancer cases worldwide in 2022, ranking second in cancer incidence. In the same year, it caused about 666,000 deaths, making it the fourth leading cause of cancer-related death. The high incidence and mortality rates have made breast cancer a major global health challenge (Wilkinson and Gathani, 2022). From the perspective of molecular classification, the hormone receptor-positive/human epidermal growth factor receptor 2-negative (HR+/HER2-) subtype is the most common. Its growth relies on the estrogen signaling pathway, accounting for approximately 70% of all breast cancer cases. Traditional treatment for this subtype primarily involves monotherapy with endocrine drugs, including selective estrogen receptor modulators and degraders. However, the frequent occurrence of drug resistance in clinical practice has severely limited patients’ long-term survival benefits (Burstein et al., 2021; Xiong et al., 2025).

Palbociclib, as the world’s first approved cyclin-dependent kinase (CDK) 4/6 inhibitor, specifically binds to the CDK4/6-cyclin D complex, blocks the phosphorylation of retinoblastoma protein, inhibits the transition of the cell cycle from the G1 phase to the S phase, and thus suppresses DNA synthesis and proliferation of tumor cells (Dhillon, 2015). Multiple key clinical trials have confirmed that palbociclib combined with letrozole or fulvestrant can significantly prolong the survival of HR+/HER2-breast cancer patients with good tolerability, and has become a first-line treatment regime (Brufsky et al., 2021; Cristofanilli et al., 2022; Rugo et al., 2022).

In clinical practice, the use of acid-reducing agents is common among cancer patients, with prevalence proportions of 20% in one major US healthcare database and 33% in another (Smelick et al., 2013). Proton pump inhibitors (PPIs) are the first choice due to their potent acid-suppressing effects and cost-effectiveness (Raoul et al., 2021). However, as a weakly basic compound, the solubility of palbociclib is pH-dependent (Chang et al., 2023; Kuminek et al., 2023). When PPIs inhibit gastric acid secretion and raise the intragastric pH above 4.5, the solubility of palbociclib drops sharply to <0.5 mg/mL, thereby significantly affecting its bioavailability (Eser et al., 2022). Studies have shown that when the palbociclib capsule formulation is co-administered with rabeprazole, under postprandial conditions, palbociclib’s C_max_ decreases by 41% and Area Under the Curve (AUC) decreases by 13%; under fasting conditions, its C_max_ decreases by 80% and AUC decreases by 62%. When taken with food, PPIs have no clinically significant impact on palbociclib exposure (Sun et al., 2017). Therefore, the capsule formulation requires administration with food (FDA, 2017). However, this is impractical for many cancer patients with poor appetite, leading to poor medication compliance and highlighting the clinical limitations of this dosage form. To address the insufficient exposure of palbociclib capsules under fasting conditions, a palbociclib tablet formulation was developed, with one of the key excipients being succinic acid. The addition of succinic acid effectively improves the environmental adaptability of the tablets, weakens the impact of intragastric pH fluctuations on drug dissolution, and ensures stable drug dissolution and good bioavailability under different intragastric environments (Zhang et al., 2019). Research results show that the exposure of the tablet is unaffected by the intragastric environment, and the prescribing information for the tablet states it can be taken with or without food (FDA, 2019a; FDA,2019b). Due to the pronounced pH-dependent pharmacokinetic (PK) properties of palbociclib (Chang et al., 2023; Kuminek et al., 2023), conventional bioequivalence (BE) studies alone may be inadequate to fully assess the equivalence between generic and original palbociclib tablets across all conditions. This is particularly critical for patients on long-term acid-reducing therapy, as such variations could directly result in significant fluctuations in clinical efficacy. To address this, the FDA mandates—in addition to standard fasting and fed BE studies—an additional BE study under fasting conditions with acid-reducing agents pre-treatment (FDA,2022). This ensures that generic versions maintain therapeutic equivalence to the original drug even in specific clinical scenarios involving acid suppression. However, due to the more intricate study design and execution required, along with higher associated costs, available data on BE studies under fasting conditions with acid-reducing agents pre-treatment remain limited. This research gap may lead to suboptimal drug exposure in clinical practice, potentially compromising efficacy and increasing the risk of treatment failure.

Recently, a generic palbociclib tablet, produced by the CSPC Ouyi Pharmaceutical Co., Ltd. (Hebei, China), is the first generic drug of its type in China. According to the National Medical Products Administration (NMPA) guidelines, this study compared the PK parameters of the new test, palbociclib tablet, with those of the reference product (Ibrance^®^). To support the marketing approval of the newly developed generic formulation in China, a pharmacokinetics and bioequivalence study was performed in healthy Chinese subjects under fasting conditions with rabeprazole pre-treatment.

## 2 Materials and methods

### 2.1 Study drugs

The acid-reducing agent was a rabeprazole enteric-coated tablet (20 mg/tablet, batch number: 2,111,129; expiry date: October 2024), which was produced by Eisai (China) Pharmaceutical Co., Ltd. under the trade name Pariet^®^ and provided by CSPC Ouyi Pharmaceutical Co., Ltd., Hebei, China. The test formulation was a palbociclib tablet, which was produced and provided by CSPC Ouyi Pharmaceutical Co., Ltd., Hebei, China (125 mg/tablet, batch number: R46220203; expiry date: 9 February 2025).The reference formulation (125 mg/tablet, batch number GT2029; expiry date: June 2025), which is marketed under the brand name Ibrance^®^ and produced by Pfizer Manufacturing Deutschland GmbH, was also provided by CSPC Ouyi Pharmaceutical Co., Ltd.

### 2.2 Ethics approval and study population

The study was registered on the Drug Clinical Trial Registration and Information Disclosure Platform [http://www.chinadrugtrials.org.cn] (number: CTR20232617, date: 23 August 2023) and retrospectively registered on the Chinese Clinical Trial Registry [https://www.chictr.org.cn] (number: ChiCTR2400084355, date: 15 May 2024). The study protocol and informed consent form were reviewed and informed consent forms (ICFs) were reviewed and approved by the independent ethics committee of Hebei General Hospital [Ethics Number: (2023) (21-01)]. The implementation of this study adhered to the ethical principles of the Declaration of Helsinki and the Guidelines for Good Clinical Practice recommended by NMPA.

This study enrolled healthy adult participants aged 18 years or older with a BMI of 19.0–26.0 kg/m^2^ (male≥50 kg, female≥45 kg) through open recruitment. The health status of all participants was confirmed through medical history review, physical examination, vital signs monitoring, 12-lead electrocardiogram (ECG), chest X-ray, and laboratory tests including complete blood count, blood biochemistry, coagulation function, urinalysis, virology screening, alcohol breath test, and drug abuse screening. Participants were excluded if they had any clinically relevant acute or chronic diseases, a history of smoking or substance abuse, an allergic constitution (particularly hypersensitivity to any component of palbociclib tablets), had donated ≥400 mL of blood or received a blood transfusion within the past 3 months, had taken investigational drugs within 3 months prior to screening, were lactating or pregnant (positive pregnancy test), or had any other factors deemed by the investigator as potentially affecting study outcomes. All participants and their partners were required to use effective contraception during the study and for 6 months after its completion. Written informed consent was obtained from all participants following a detailed explanation of the study’s purpose, content, procedures, and potential risks.

All participants signed the written informed consent form after having sufficient time to review the document and discuss any questions with the study staff. They were informed that they were free to withdraw from the study at any time. The generation of the random allocation sequence was solely managed and kept strictly confidential by the Principal Investigator. Throughout the study period, internists were responsible for the continuous medical supervision of the participants, monitoring medication safety, and managing adverse events (AEs). All AEs were documented in detail and promptly addressed, with monitoring and follow-up continuing throughout the entire study period until resolution, return to baseline, or stabilization to a level deemed acceptable by the investigators. Furthermore, the study would be terminated prematurely in the event of any of the following circumstances, including but not limited to: serious adverse events (SAEs) related to the investigational drug, major protocol deviations that significantly affected the study outcomes, or a termination order issued by the NMPA or the ethics committee.

### 2.3 Study design

**FIGURE 1:**
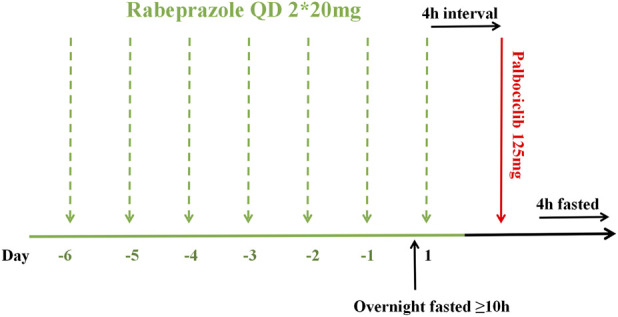
Dosing schedule timeline.

This was a single-center, randomized, open-label, single-dose, two-period crossover bioequivalence study conducted under fasting conditions with rabeprazole pre-treatment. It was carried out at the Phase I Clinical Research Center of Hebei General Hospital in Shijiazhuang, Hebei Province, China, from 12 September 2023, to 27 October 2023. Using SAS statistical software (version 9.4), subjects were randomly allocated in a 1:1 ratio to either the T-R or R-T sequence (where T represents the test formulation and R denotes the reference formulation), with a 14-day washout period justified by palbociclib’s half-life of 29 ± 5 h (FDA.,2019), and exceeding seven elimination half-lives between the two treatment periods. As shown in Figure 1, all subjects received 40 mg oral rabeprazole enteric-coated tablets (2 × 20 mg) with 240 mL water once daily approximately 30 min before breakfast from Day −6 to Day −1. Water intake was prohibited from 1 h before to 1 h after each dose (except for breakfast and water used for medication administration). On Day 1, under fasted conditions (at least 10 h), subjects received their seventh dose of rabeprazole and maintained fasting for an additional 4 h. They then took a single 125 mg dose (1 tablet) of either the test or reference formulation orally, according to the randomized sequence, with 240 mL of water. Water intake was prohibited from 1 h before to 1 h after administration. Standardized meals were provided at 4 and 10 h post-dose.

Previous studies have demonstrated that palbociclib exhibits an intra-individual coefficient of variation (Intra-CV) of approximately 26% (FDA.,2019). This study utilized AUC and C_max_ as primary pharmacokinetic parameters. Assuming a one-sided test with α = 0.05, power of 0.8 (β = 0.2), and Intra-CV = 25%. The anticipated geometric mean ratio between test and reference formulations was 0.95–1.05, with bioequivalence acceptance criteria set at 80.00%–125.00%. Based on these parameters, the calculated sample size requirement was 28 evaluable subjects. To account for potential dropouts, the study planned to enroll 36 participants.

### 2.4 Blood sampling and bioanalytical assay

In this study, serial blood samples for palbociclib pharmacokinetic analysis were collected at baseline (1 h before dosing) and at 1.0, 2.0, 3.0, 4.0, 5.0, 6.0, 7.0, 8.0, 9.0, 10.0, 11.0, 12.0, 24.0, 48.0, 72.0, and 96.0 h after dosing under fasting conditions with rabeprazole pre-treatment. Blood samples were centrifuged at 1700 *g* for 10 min at 2 °C–8 °C within 60 min of collection to obtain plasma supernatant, which was then stored at −60 °C until analysis. Plasma concentrations of palbociclib were quantified using a validated high-performance liquid chromatography-tandem mass spectrometry (HPLC-MS/MS) method at a specialized analytical laboratory (Nanjing Kelitai Pharmaceutical Technology Co., Ltd., Nanjing, China), in compliance with both China NMPA and FDA guidelines.

### 2.5 Pharmacokinetic analysis

The primary pharmacokinetic endpoints for palbociclib were C_max_, AUC_0-t_ and ${\text{AUC}}_{0‐\infty }$ . T_max_, t_1/2z_, elimination rate constant (λ_z_) and percentage of extrapolated residual area (AUC__%Extrap_) were the secondary endpoints. Based on the plasma concentration data, the pharmacokinetic parameters of palbociclib were calculated using a non-compartmental model using Phoenix WinNonlin software (version 8.3). C_max_ and T_max_ were determined directly from the observed plasma concentration-time profiles. AUC_0-t_ was calculated using the linear/log trapezoidal method. ${\text{AUC}}_{0‐\infty }$ was determined by formula: ${\text{AUC}}_{0‐\infty }$ = AUC_0-t_ + C_t_/λ_z_, where C_t_ was the last measurable concentration. The slope of the terminal segment of the semi-logarithmic drug-time curve calculated by linear regression is used to obtain λ_z_. t_1/2z_ was calculated as ln2/λ_z_. AUC__%Extrap_ was determined by formula: AUC__%Extrap_ = [( ${\text{AUC}}_{0‐\infty }$ - AUC_0-t_)/ ${\text{AUC}}_{0‐\infty }$ ]×100%. For pharmacokinetic parameters that cannot be calculated due to inapplicable data, they are marked with “-” in the dataset.

### 2.6 Bioequivalence and statistical analysis

After log-transforming C_max_, AUC_0−t_, and ${\text{AUC}}_{0‐\infty }$ , analysis of variance (using the PROC MIXED method) was performed via a mixed linear effects model, with formulation, period, and sequence treated as fixed effects and subject nested within sequence as a random effect. Throughout the analyses, missing data were, in principle, not imputed. Bioequivalence was assessed using the two one-sided t-tests, and the 90% confidence interval (CI) for the ratio of the geometric least squares means of the two formulations was calculated. If the CI fell entirely within the range of 80.00%–125.00%, the formulations were deemed bioequivalent. Tmax was evaluated using the non-parametric Wilcoxon signed-rank test, and statistical significance was considered when P < 0.05. Pharmacokinetic parameters were calculated using Phoenix WinNonlin (version 8.3), while the remaining statistical analyses and data processing were performed by SAS software (version 9.4). Continuous variables were presented with descriptive statistics, and categorical data were expressed as frequencies and percentages.

### 2.7 Safety assessment

Safety assessments included AEs, SAEs, concomitant medications, laboratory tests, clinical symptoms, vital sign measurements, 12-lead ECG and physical examination results. Seated vital signs of subjects (axillary temperature, pulse and blood pressure) were measured within 1 h before and within 1 h after taking rabeprazole each time, as well as within 1 h before and at 2.0 ± 0.5, 6.0 ± 0.5, 24.0 ± 1.0, 48.0 ± 1.0, 72.0 ± 1.0and 96.0 ± 1.0 h after taking palbociclib. Laboratory tests, physical examinations, and 12-lead ECG were performed at screening and during the follow-up period. All AEs were monitored throughout the study by the research doctors and spontaneously reported by the subjects. AEs were coded using MedDRA (Medical Dictionary for Regulatory Activities: Version 26.1) and summarized according to System Organ Class (SOC) and Preferred Term (PT), including the number and percentage of subjects experiencing AEs and the frequency of AEs. The severity of AEs was graded according to the Common Terminology Criteria for Adverse Events (CTCAE, Version 5.0) issued by the U.S. National Cancer Institute. Missing safety data were labeled as “Missing” in the datasets and were not imputed in the subsequent analyses.

## 3 Results

### 3.1 Study population

**FIGURE 2:**
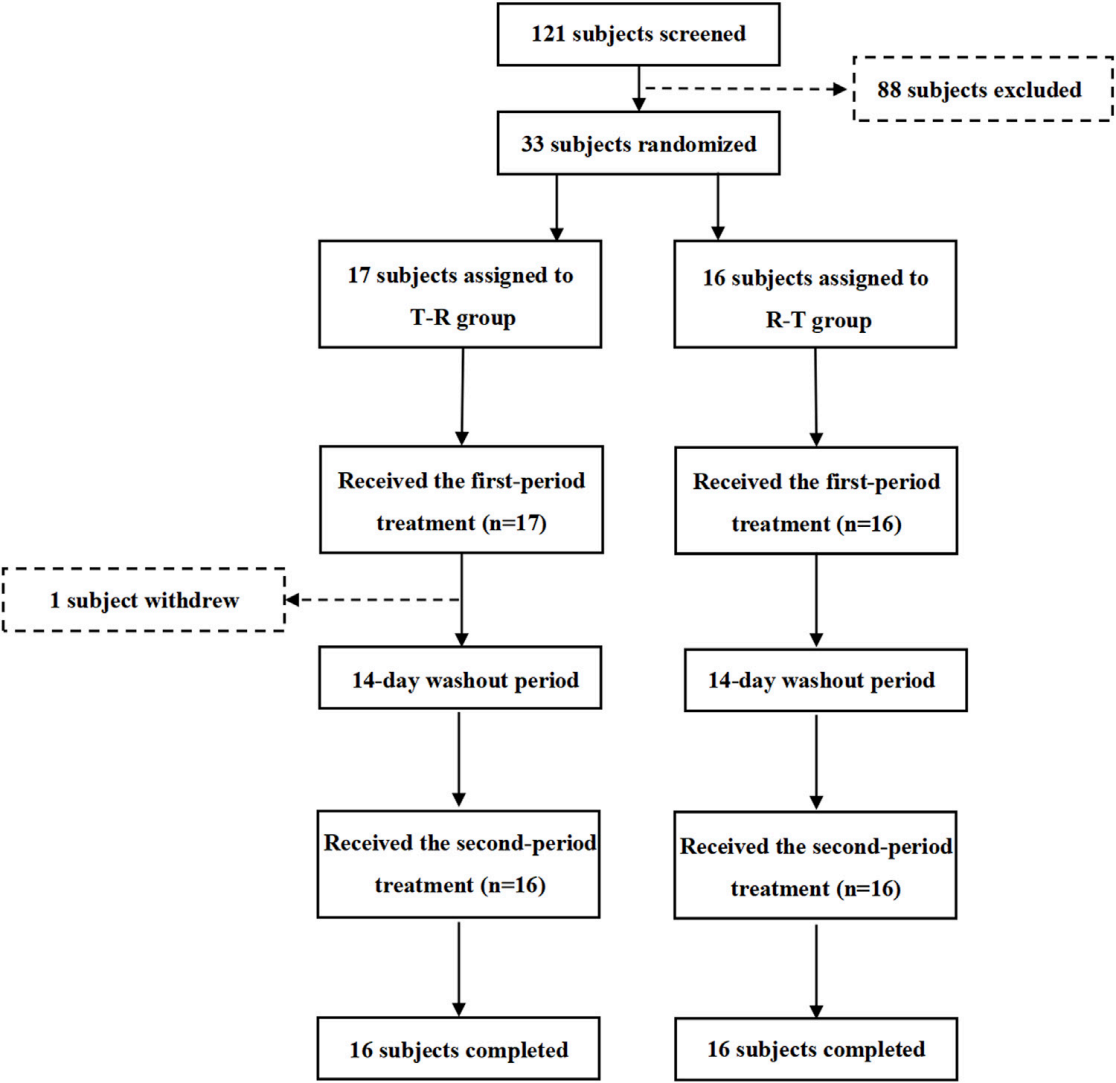
Study design and disposition of subjects.

The flow of participant screening, enrollment, and study completion is summarized in Figure 2. A total of 121 potential Chinese adults were screened. Of these, 33 healthy subjects met the eligibility criteria for the protocol and were enrolled and randomized, with 17 subjects assigned to the T-R group and 16 subjects to the R-T group. Table 1 summarizes the demographic characteristics of all subjects. The age, sex, weight, height, BMI, and race of the subjects were similar between the two parts of the study. In the T-R group, one subject (Y004) withdrew for personal reasons after the end of the first cycle, and the remaining 32 subjects completed both periods.

**TABLE 1:** Demographic characteristics of the healthy subjects.

Parameters	T-R	R-T	Total
N	17	16	33
Age (years)			
Mean ± SD	33.5 ± 8.60	34.4 ± 9.56	33.9 ± 8.94
Min ∼ Max	20–47	22–51	20–51
Gender *n* (%)			
Male	15 (88.20)	14 (87.50)	29 (87.90)
Female	2 (12.50)	2 (12.50)	4 (12.10)
Weight (kg)			
Mean ± SD	65.76 ± 7.179	64.18 ± 7.518	65.00 ± 7.274
Min ∼ Max	48.1–76.1	53.0–76.8	48.1–76.8
Height (cm)			
Mean ± SD	167.44 ± 6.576	167.59 ± 6.328	167.52 ± 6.356
Min ∼ Max	156.0–177.5	157.0–180.5	156.0–180.5
BMI (kg/m^2^)			
Mean ± SD	23.43 ± 1.918	22.83 ± 2.121	23.14 ± 2.010
Min ∼ Max	19.8–25.9	19.5–26.0	19.5–26.0
Ethnicity *n* (%)			
Ethnic Han	17 (100)	15 (93.8)	32 (97.0)
Others	0 (0)	1 (6.3)	1 (3.0)

Abbreviations: BMI, body mass index; SD, standard deviation.

### 3.2 Pharmacokinetics results

**FIGURE 3:**
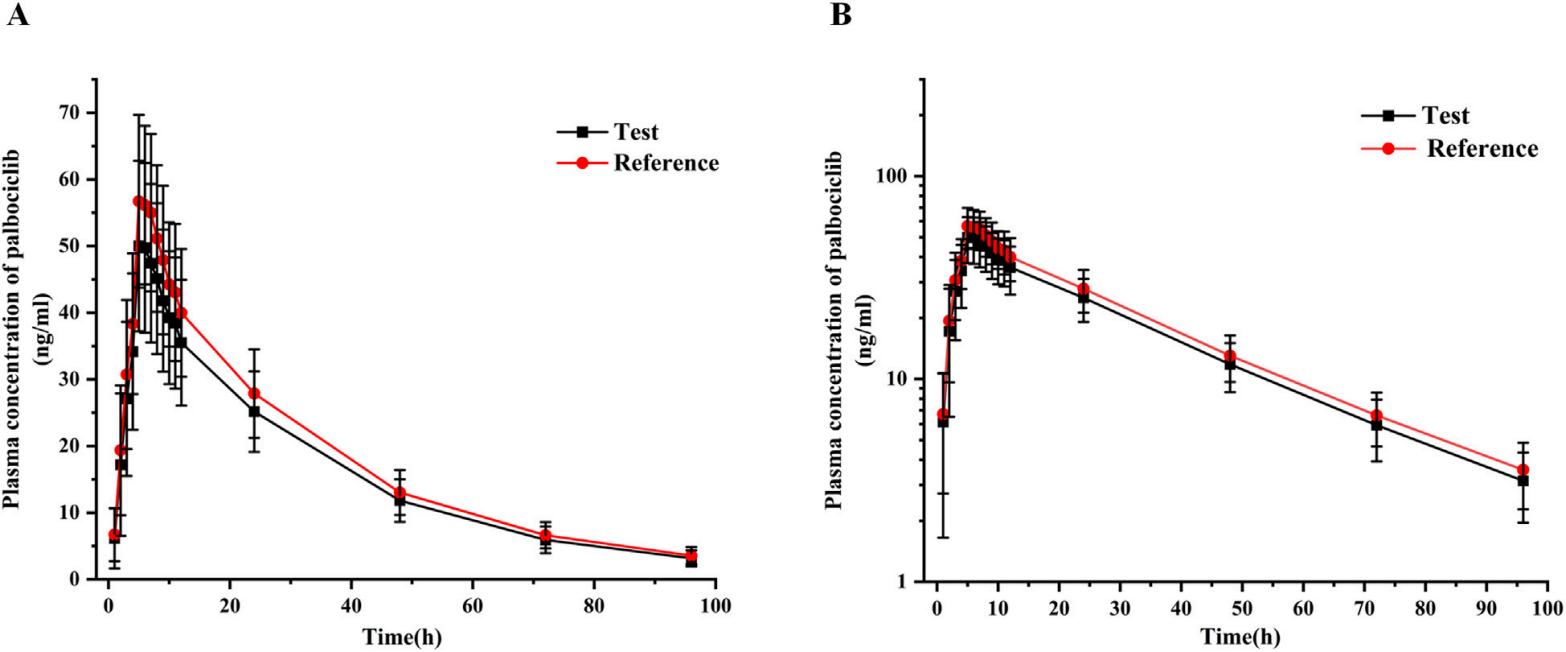
Mean plasma concentration-time curves of test and reference palbociclib (125 mg) under fasting conditions with rabeprazole pre-treatment. **(A)** Linear-scale profile; **(B)** Semi-logarithmic-scale profile. Data are presented as mean ± SD.

All PK parameters were analyzed based on the pharmacokinetics concentration set (PKCS) and pharmacokinetics parameter set (PKPS). In the T-R group, one subject withdrew during the second cycle, and only the data from the first cycle of this subject were included in PKCS and PKPS. Therefore, in the PK analysis, 33 datasets were included for the test formulation, and 32 datasets were included for the reference formulation. The major PK parameters of palbociclib were calculated using a non-compartmental model and are summarized in Table 2.The results showed that a C_max_ value of 51.68 ± 12.96 ng/mL (CV% 25.07) of palbociclib was achieved within 5.0–7.0 h after oral administration of the test palbociclib tablet; the mean AUC_0-t_ and ${\text{AUC}}_{0‐\infty }$ value was 1543.38 ± 365.51 h·ng/mL (CV% 23.68) and 1659.93 ± 394.87 h·ng/mL (CV% 23.79), respectively. Palbociclib was eliminated very slowly, with a terminal elimination half-life (t_1/2z_)of 24.48 ± 3.98 h (CV% 16.26). After oral administration of the reference palbociclib tablet under fasting conditions, a C_max_ value of 59.05 ± 12.66 ng/mL (CV% 21.43) of palbociclib was achieved within 5.0–8.0 h; the mean AUC_0-t_ and ${\text{AUC}}_{0‐\infty }$ value was 1724.85 ± 373.25 h·ng/mL (CV%21.64) and 1860.11 ± 402.10 h·ng/mL (CV% 21.62), respectively. The reference palbociclib also exhibited a slow elimination profile, with a terminal elimination half-life of 25.32 ± 4.58 h (CV% 18.09). These results indicated that the PK parameters of the test formulation were similar to those of the reference formulation. As shown in Figure 3, the mean plasma concentration-time curves of the test and reference products under fasting conditions with rabeprazole pre-treatment were consistent. These results suggest that the *in vivo* disposal process of the test product was similar to that of the reference product under fasting conditions with rabeprazole pre-treatment.

**TABLE 2:** Pharmacokinetic parameters.

Parameter	Mean ± SD (CV%)	
	Test (*N* = 33)	References (*N* = 32)
C_max_ (ng/mL)	51.68 ± 12.96 (25.07)	59.05 ± 12.66 (21.43)
T_max_ (h)	5.99 (5.0,7.0)	5.99 (5.0,8.0)
AUC_0-t_ (h·ng/mL)	1543.38 ± 365.51 (23.68)	1724.85 ± 373.25 (21.64)
${\text{AUC}}_{0‐\infty }$ (h·ng/mL)	1659.93 ± 394.87 (23.79)	1860.11 ± 402.10 (21.62)
t_1/2z_ (h)	24.48 ± 3.98 (16.26)	25.32 ± 4.58 (18.09)
λ_z_ (1/h)	0.029 ± 0.0046 (15.83)	0.028 ± 0.0046 (16.30)
AUC__%Extrap_ (%)	6.36 (2.6,13.7)	6.41 (2.8,15.3)

Values are expressed as mean ± SD, except Tmax and AUC__%Extrap_, which is the median (min, max).

Abbreviations: C_max_, maximum plasma concentration; Tmax, time to Cmax; AUC_0-t_, area under the concentration curve from 0 time to t; ${\text{AUC}}_{0‐\infty }$ , area under the concentration curve from 0 time to infinity; t_1/2z_, terminal elimination half-life; λ_z_, the elimination rate constant; AUC__%Extrap_, the percentage of ${\text{AUC}}_{0‐\infty }$ contributed by extrapolation; SD, standard deviation; CV, coefficient of variation.

### 3.3 Bioequivalence results

For PK bioequivalence evaluation, the subjects were enrolled in the bioequivalence analysis set (BES) as with PKPS under fasting conditions with rabeprazole pre-treatment. As shown in Table 3, the coefficient of intra-subject variation (CV) of C_max_, AUC_0-t_ and ${\text{AUC}}_{0‐\infty }$ were 9.65%, 6.14% and 5.83%, respectively, all less than 10%. The average bioequivalence criterion was adopted to evaluate the bioequivalence PK parameters. The GLSM ratios of the test to reference products for C_max_, AUC_0-t_ and ${\text{AUC}}_{0‐\infty }$ were 88.05%, 90.12% and 89.78%, respectively. The corresponding 90% CIs for C_max_, AUC_0-t_ and ${\text{AUC}}_{0‐\infty }$ were 84.53%–91.72%, 87.81%–92.49% and 87.59%–92.03%, respectively, all within the accepted bioequivalence range of 80.00%–125.00%, indicating that the test and reference products of palbociclib were bioequivalent under fasting conditions with rabeprazole pre-treatment. The Wilcoxon signed-rank test (P = 0.0699) also indicated that there was no significant difference in Tmax between the two products.

**TABLE 3:** Bioequivalence assessment of the primary pharmacokinetic parameters.

PK parameter	GLSM		Ratio (%)	Intra-subject CV (%)	Power (%)	90% CI (%)
	Test (n = 33)	References (n = 32)				
C_max_ (ng/mL)	50.21	57.02	88.05	9.65	99.0	84.53–91.72
AUC_0-t_ (h·ng/mL)	1505.54	1670.59	90.12	6.14	100.0	87.81–92.49
${\text{AUC}}_{0‐\infty }$ (h·ng/mL)	1618.48	1802.65	89.78	5.83	100.0	87.59–92.03

Abbreviations: C_max_, maximum plasma concentration; AUC_0-t_, area under the concentration curve from 0 time to t; ${\text{AUC}}_{0‐\infty }$ , area under the concentration curve from 0 time to infinity; GLSM, geometric least-squares mean; CV,coefficient of variation; CI, confidence intervals.

To assess whether period, sequence, and formulation factors had an impact on the current study, a multivariate analysis of variance was performed using a linear mixed-effects model for the natural logarithmic of C_max_, AUC_0-t_ and ${\text{AUC}}_{0‐\infty }$ in the study, respectively. The results showd that, except for the significant differences in the effects of formulation on all three parameters and the significant differences in the effects of administration sequence on AUC_0-t_ and ${\text{AUC}}_{0‐\infty }$ , there were no significant differences in other factors. However, the establishment of bioequivalence was not affected.

### 3.4 Safety assessment

The safety and tolerability of palbociclib were assessed based on the safety analysis set (SS).In this study, 33 subjects received at least one dose of the study drug and were included in SS. Twenty-eight AEs for 17 subjects were reported, and the incidence of AEs was 51.5% (17/33) (Table 4). Of these, 15 AEs were reported for 11 subjects in the test product; the incidence of AEs was 33.3% (11/33) (Table 4), whereas 13 AEs were reported for nine subjects and an incidence of 28.1% (9/32) in the reference product (Table 4). Among the AEs related to the test product, two subjects experienced serum thyroid-stimulating hormone (TSH) increased and nausea, respectively, which were defined as adverse drug reactions (ADRs). Two AEs for two subjects related to the reference product were defined as ADRs, including hemoglobin decreased and urinary tract infection, respectively. All AEs were mild and reported as grade 1. No SAEs occurred during the study. All AEs or ADRs were spontaneously resolved without any specific treatment. The results indicated that palbociclib had good safety and was well tolerated in healthy subjects under fasting conditions with rabeprazole pre-treatment.

**TABLE 4:** Summary of treatment-emergent AEs.

Parameter	Test (n = 33)		References (n = 32)	
	AE count	N (%)	AE count	N (%)
Sum	15	11 (33.3)	13	9 (28.1)
Grade 1	15	11 (33.3)	13	9 (28.1)
Grade 2	0	0 (0)	0	0 (0)
≥ Grade 3	0	0 (0)	0	0 (0)
Results of inspection	13	10 (30.3)	11	8 (25.0)
Blood pressure increased	4	3 (9.1)	4	4 (12.5)
TG increased	3	3 (9.1)	1	1 (3.1)
Conjugated bilirubin increased	1	1 (3.0)	1	1 (3.1)
Bilirubin increased	1	1 (3.0)	1	1 (3.1)
TSH increased	1	1 (3.0)	0	0 (0)
Blood phosphorus increased	1	1 (3.0)	0	0 (0)
ECG T-wave abnormalities	1	1 (3.0)	0	0 (0)
ECG ST segment sepression	1	1 (3.0)	0	0 (0)
SCr increased	0	0 (0)	2	2 (6.3)
Hemoglobin decreased	0	0 (0)	1	1 (3.1)
Neutrophil count increased	0	0 (0)	1	1 (3.1)
Infections and infestations	0	0 (0)	1	1 (3.1)
Urinary tract infection	0	0 (0)	1	1 (3.1)
Gastrointestinal disorders	1	1 (3.0)	0	0 (0)
Nausea	1	1 (3.0)	0	0 (0)
Skin and subcutaneous tissue	1	1 (3.0)	1	1 (3.1)
Allergic dermatitis	0	0 (0)	1	1 (3.1)
Hyperhidrosis	1	1 (3.0)	0	0 (0)

Abbreviations: TG, triglyceride; TSH, thyroid-stimulating hormone; ECG, electrocardiogram; SCr, serum creatinine.

## 4 Discussion

This study adheres to the guidelines issued by the NMPA, providing evidence of bioequivalence between generic and original palbociclib tablets in healthy Chinese subjects under fasting conditions with rabeprazole pre-treatment. Both formulations met pharmacokinetic bioequivalence criteria and were well-tolerated, offering substantial basis for understanding the drug’s pharmacokinetic characteristics in Chinese populations and supporting abbreviated new drug application approvals.

Palbociclib is categorized as a Biopharmaceutics Classification System (BCS) Class II drug, characterized by low solubility and high intestinal permeability. Drugs within this class often exhibit a greater likelihood of demonstrating food-dependent pharmacokinetic variations (Chu et al., 2022; Kuminek et al., 2023). The FDA mandates fasting, postprandial, and acid-reducing agent pre-treatment bioequivalence studies (FDA, 2022). This trial employed a single-dose, two-period crossover design under fasting conditions with rabeprazole pre-treatment, with a 14-day washout period to ensure drug elimination given palbociclib’s 29 ± 5-h half-life (FDA, 2019a). For pharmacokinetic research, a scientifically validated sampling design holds critical importance, as it necessitates a sample size capable of fully capturing the plasma concentration-time profile to ensure accurate exposure assessment. This benchmark is fulfilled when the AUC_0-t_ constitutes over 80% of the ${\text{AUC}}_{0‐\infty }$ (EMA, 2010). This study confirms that the sampling protocol at 96 h after palbociclib administration can make AUC_0-t_ cover 93% of ${\text{AUC}}_{0‐\infty }$ , which has outstanding advantages in improving subject compliance and research efficiency.

To evaluate the most extreme impact of PPIs on palbociclib PK, rabeprazole was chosen as the PPI due to its superior 24-h median intragastric pH, quicker onset of action, and prolonged acid suppression duration compared with other PPIs (EMA, 2010; Smelick et al., 2013). Additionally, rabeprazole is almost completely metabolized via non-enzymatic metabolic pathways, with metabolites excreted through the kidneys. Minor enzyme-mediated metabolism involves CYP3A4 and CYP2C1922 (Pace et al., 2007; Swan et al., 1999). Its metabolic pathways, primarily via CYP3A and SULT2A1 for palbociclib (FDA, 2019a), show no significant overlap, thus avoiding drug-drug interactions.

The pharmacokinetic characteristics as we showed in this study, were compared with capsules in healthy Chinese subjects under fed conditions (Chu et al., 2022). The results showed that the AUC_0-t_ of the tablets was 1543.38 h·ng/mL, representing a 4% decrease compared with the capsule’s 1608.10 h·ng/mL. Meanwhile, the C_max_ (51.68 ng/mL vs. 57.6 ng/mL) and ${\text{AUC}}_{0‐\infty }$ (1659.932 h·ng/mL vs. 1642.26 h·ng/mL) exhibited minimal differences between the two formulations. This confirms that the palbociclib tablets formulated with succinic acid effectively mitigate the bioavailability reduction typically induced by elevated gastric pH conditions, which is consistent with FDA findings (FDA, 2019a; FDA, 2019b).

The safety analysis showed that a total of 17 subjects reported 28 adverse events, among which 4 were confirmed as adverse reactions to palbociclib. Neutropenia represents the most frequent adverse drug reaction associated with CDK4/6 inhibitor class agents (FDA, 2019a; Finn et al., 2015). The underlying mechanism involves blockade of the CDK4/6-Rb pathway, which induces cell cycle arrest (G1 phase) in early bone marrow progenitor cells, consequently impairing neutrophil differentiation and maturation (FDA, 2019a; Dhillon, 2015; Johnson et al., 2010). Although no typical neutropenic events were observed in this study, the reported hemoglobin reduction may be attributed to analogous inhibitory effects on erythroid progenitors, a finding consistent with palbociclib’s established myelosuppressive profile.The increase in serum thyroid-stimulating hormone (TSH) may be attributed to palbociclib interfering with the proliferation cycle of thyroid follicular cells, causing G1-S phase arrest, which indirectly affects the synthesis and feedback regulation of thyroid hormones, leading to TSH elevation (FDA, 2019b; Dhillon, 2015). Nausea is associated with direct stimulation of the gastric mucosa by drug dissolution after fasting administration and activation of intestinal 5-HT_3_ receptors. The mechanism of urinary tract infection has not been fully elucidated. Although succinic acid was added to optimize the tablet formulation used in this study, the types and incidence of drug adverse reactions observed were basically consistent with those of the capsule formulation (FDA, 2017), and all events were mild, indicating that the palbociclib tablet has good overall tolerance in healthy humans.

## 5 Conclusion

According to the results of the current study, the generic palbociclib tablets showed bioequivalence with the reference preparation (Ibrance^®^) in terms of the extent and rate of absorption under fasting conditions with rabeprazole pre-treatment, and the bioavailability of the test preparation was similar to that of the reference preparation. Both formulations are generally well-tolerated in healthy Chinese populations and can be used interchangeably in clinical practice.

## Data Availability

The raw data supporting the conclusions of this article will be made available by the authors, without undue reservation.

## References

[B1] Brufsky A. Liu X. Li B. McRoy L. Layman R. M. (2021). Real-world tumor response of palbociclib plus letrozole *versus* letrozole for metastatic breast cancer in US clinical practice. Target Oncol. 16 (5), 601–611. 10.1007/s11523-021-00826-1 34338965 PMC8484164

[B2] Burstein H. J. Curigliano G. Thürlimann B. Weber W. P. Poortmans P. Regan M. M. (2021). Customizing local and systemic therapies for women with early breast cancer: the St. Gallen International consensus Guidelines for treatment of early breast cancer 2021. Ann. Oncol. 32 (10), 1216–1235. 10.1016/j.annonc.2021.06.023 34242744 PMC9906308

[B3] Chang Y. C. Song J. Chang Y. Huang C. H. Sudan A. Chen P. C. (2023). The Association between proton pump inhibitors and the effectiveness of CDK inhibitors in HR+/HER- advanced breast cancer patients: a systematic review and meta-analysis. Cancers (Basel) 15 (21), 5133. 10.3390/cancers15215133 37958308 PMC10649865

[B4] Chu N. N. Zhang L. Wang J. Gu X. Ding Y. Huang K. (2022). Bioequivalence Study of Palbociclib capsules in healthy Chinese subjects under fasting and Fed conditions. Clin. Drug Investig. 42 (1), 53–63. 10.1007/s40261-021-01103-9 34837169

[B5] Cristofanilli M. Rugo H. S. Im S. A. Slamon D. J. Harbeck N. Bondarenko I. (2022). Overall survival with palbociclib and fulvestrant in women with HR+/HER2- ABC: updated exploratory analyses of PALOMA-3, a double-blind, phase III randomized Study. Clin. Cancer Res. 28 (16), 3433–3442. 10.1158/1078-0432.Ccr-22-0305 35552673 PMC9662922

[B6] Dhillon S. (2015). Palbociclib: first global approval. Drugs 75 (5), 543–551. 10.1007/s40265-015-0379-9 25792301

[B7] EMA (2010). Guideline on the investigation of bioequivalence. Available online at: https://www.gmp-compliance.org/files/guidemgr/2016_EMEA_Bioequivalence.pdf (Accessed May 16, 2025).

[B8] Eser K. Önder A. H. Sezer E. Çil T. İnal A. Öztürk B. (2022). Proton pump inhibitors may reduce the efficacy of ribociclib and palbociclib in metastatic breast cancer patients based on an observational study. BMC Cancer 22 (1), 516. 10.1186/s12885-022-09624-y 35525929 PMC9078089

[B9] FDA (2017). Highlights of Prescribing Information for IBRANCE^®^ (palbociclib) capsules, for oral use. Available online at: https://www.accessdata.fda.gov/drugsatfda_docs/label/2017/207103s004lbl.pdf (Accessed May 15.

[B10] FDA (2019a). Clinical review for application 212436Orig1s000. Available online at: https://www.accessdata.fda.gov/drugsatfda_docs/nda/2019/212436Orig1s000ClinPharmR.pdf (Accessed May 15, 2025).

[B11] FDA (2019b). Highlights of Prescribing Information for IBRANCE^®^ (palbociclib) tablets, for oral use. Available online at: https://www.accessdata.fda.gov/drugsatfda_docs/label/2019/212436lbl.pdf (Accessed May 15, 2025).

[B12] FDA (2022). Draft guidance on palbociclib. Available online at: https://www.accessdata.fda.gov/drugsatfda_docs/psg/PSG_212436.pdf (Accessed May 16, 2025).

[B13] Finn R. S. Crown J. P. Lang I. Boer K. Bondarenko I. M. Kulyk S. O. (2015). The cyclin-dependent kinase 4/6 inhibitor palbociclib in combination with letrozole *versus* letrozole alone as first-line treatment of oestrogen receptor-positive, HER2-negative, advanced breast cancer (PALOMA-1/TRIO-18): a randomised phase 2 study. Lancet Oncol. 16 (1), 25–35. 10.1016/s1470-2045(14)71159-3 25524798

[B14] Johnson S. M. Torrice C. D. Bell J. F. Monahan K. B. Jiang Q. Wang Y. (2010). Mitigation of hematologic radiation toxicity in mice through pharmacological quiescence induced by CDK4/6 inhibition. J. Clin. Invest 120 (7), 2528–2536. 10.1172/jci41402 20577054 PMC2898594

[B15] Kuminek G. Salehi N. Waltz N. M. Sperry D. C. Greenwood D. E. Hate S. S. (2023). Use of gastrointestinal simulator, mass transport analysis, and absorption simulation to investigate the impact of pH modifiers in mitigating weakly basic drugs' performance issues related to gastric pH: palbociclib case Study. Mol. Pharm. 20 (1), 147–158. 10.1021/acs.molpharmaceut.2c00545 36367432

[B16] Pace F. Pallotta S. Casalini S. Porro G. B. (2007). A review of rabeprazole in the treatment of acid-related diseases. Ther. Clin. Risk Manag. 3 (3), 363–379. 18488081 PMC2386363

[B17] Raoul J. L. Guérin-Charbonnel C. Edeline J. Simmet V. Gilabert M. Frenel J. S. (2021). Prevalence of proton pump inhibitor use among patients with cancer. JAMA Netw. Open 4 (6), e2113739. 10.1001/jamanetworkopen.2021.13739 34132796 PMC8209575

[B18] Rugo H. S. Brufsky A. Liu X. Li B. McRoy L. Chen C. (2022). Real-world study of overall survival with palbociclib plus aromatase inhibitor in HR+/HER2-metastatic breast cancer. NPJ Breast Cancer 8 (1), 114. 10.1038/s41523-022-00479-x 36220852 PMC9553912

[B19] Smelick G. S. Heffron T. P. Chu L. Dean B. West D. A. Duvall S. L. (2013). Prevalence of acid-reducing agents (ARA) in cancer populations and ARA drug-drug interaction potential for molecular targeted agents in clinical development. Mol. Pharm. 10 (11), 4055–4062. 10.1021/mp400403s 24044612

[B20] Sun W. Klamerus K. J. Yuhas L. M. Pawlak S. Plotka A. O'Gorman M. (2017). Impact of acid-reducing agents on the pharmacokinetics of Palbociclib, a weak base with pH-Dependent solubility, with different food intake conditions. Clin. Pharmacol. Drug Dev. 6 (6), 614–626. 10.1002/cpdd.356 28430398

[B21] Swan S. K. Hoyumpa A. M. Merritt G. J. (1999). Review article: the pharmacokinetics of rabeprazole in health and disease. Aliment. Pharmacol. Ther. 13 (Suppl. 3), 11–17. 10.1046/j.1365-2036.1999.00020.x 10491724

[B22] Wilkinson L. Gathani T. (2022). Understanding breast cancer as a global health concern. Br. J. Radiol. 95 (1130), 20211033. 10.1259/bjr.20211033 34905391 PMC8822551

[B23] Xiong X. Zheng L. W. Ding Y. Chen Y. F. Cai Y. W. Wang L. P. (2025). Breast cancer: pathogenesis and treatments. Signal Transduct. Target Ther. 10 (1), 49. 10.1038/s41392-024-02108-4 39966355 PMC11836418

[B24] Zhang M. Xiong X. Suo Z. Hou Q. Gan N. Tang P. (2019). Co-amorphous palbociclib-organic acid systems with increased dissolution rate, enhanced physical stability and equivalent biosafety. RSC Adv. 9 (7), 3946–3955. 10.1039/c8ra09710k 35518078 PMC9060427

